# TREX1, a predator for treating MSI‐H tumors?

**DOI:** 10.1002/1878-0261.70173

**Published:** 2025-11-25

**Authors:** Elena Benidovskaya, Joséphine Deneft, Marc Van den Eynde

**Affiliations:** ^1^ IREC MIRO‐ONCO Laboratory of Oncology, UCLouvain Brussels Belgium; ^2^ Medical Oncology and Gastroenterology Department Cliniques Universitaires Saint‐Luc Brussels Belgium; ^3^ Institut Roi Albert II, Cliniques Universitaires Saint‐Luc Brussels Belgium

**Keywords:** cGAS‐STING pathway, immune escape, immunotherapy, MSI‐H tumors, TREX1 exonuclease

## Abstract

Immunotherapy has revolutionized cancer treatment; yet, a subset of patients with microsatellite instability‐high (MSI‐H) tumors fails to respond to treatment despite their elevated tumor mutational burden and immunogenic potential. In a recent study, Xu *et al*. uncover a key mechanism of immune evasion in MSI‐H tumors mediated by the exonuclease TREX1, which degrades cytosolic DNA and suppresses activation of the cyclic GMP‐AMP synthase—stimulator of interferon genes (cGAS‐STING)—type I interferon pathway. Loss of TREX1 restores cytosolic DNA sensing, promotes CD8^+^ T and NK cell infiltration, and enhances antitumor immunity. These findings highlight TREX1 as a potential therapeutic target to overcome resistance to immune checkpoint blockade.

## Graphical abstract

Immunotherapy benefits many patients; yet, some with MSI‐H tumors remain unresponsive despite their high immunogenicity. Xu *et al*. reveal that TREX1 enables immune evasion by degrading cytosolic DNA and suppressing cGAS–STING–IFN‐I signaling. TREX1 loss restores DNA sensing, increases CD8^+^ T and NK cell infiltration, and boosts antitumor immunity, highlighting TREX1 as a promising target to overcome immunotherapy resistance.

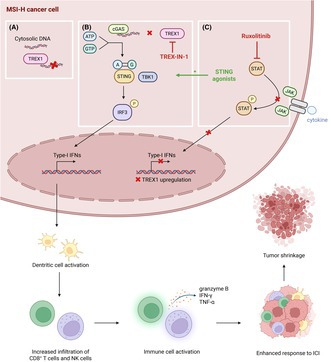

**Abbreviations**
cGAS: cyclic GMP‐AMP synthase
CTLA‐4: cytolytic T‐lymphocyte associated protein 4
DC: dendritic cells
ICI: immune checkpoint inhibitors
IFN‐I: type I interferon
MSI‐H: microsatellite instability—high
MSS: microsatellite‐stable
NK: natural killer cells
PD‐1: programmed death protein 1
PD‐L1: programmed death‐ligand 1
STING: stimulator of interferon genes
TMB: tumor mutational burden
TREX1: three‐prime repair exonuclease 1
TREX1‐IN‐1: TREX 1 inhibitor

In recent years, immunotherapy has emerged as a promising therapeutic strategy in oncology, offering durable clinical responses in several cancer types by harnessing the body's immune system to target cancer cells. Among these approaches, immune checkpoint inhibitors (ICI), targeting molecules such as programmed death protein 1 (PD‐1), programmed death‐ligand 1 (PD‐L1), or cytolytic T‐lymphocyte associated protein 4 (CTLA‐4), have shown remarkable efficacy in tumors with high levels of immune infiltration and genomic instability [1].

Patients with high microsatellite instability (MSI‐H) tumors demonstrate significantly improved responses to immunotherapy and overall survival compared to patients with microsatellite‐stable (MSS) tumors [2]. This improved sensitivity is largely attributed to the elevated tumor mutational burden (TMB) characteristic of MSI‐H tumors, which leads to the generation of numerous neoantigens capable of eliciting robust antitumor immune responses [3].

However, despite their immunogenic potential, a subset of MSI‐H patients fails to benefit from immunotherapy. The mechanisms underlying this resistance remain poorly understood, suggesting that factors beyond TMB, such as the composition and functional state of tumor‐infiltrating and circulating immune cells, may play critical roles in modulating treatment efficacy. Understanding these immune determinants is essential to identify biomarkers predictive of response and to optimize patient selection for immunotherapy [4].

To overcome this limitation, various strategies are currently being explored to enhance the immunogenicity of these tumors and improve patient outcomes. These include combination approaches integrating immunotherapy with chemotherapy, radiotherapy, targeted agents, or novel immunomodulatory treatments aimed at reshaping the tumor microenvironment [5, 6, 7].

In this context, Xu *et al*. investigated the mechanisms by which MSI‐H tumors might evade immune surveillance [8]. They proposed that three‐prime repair exonuclease 1 (TREX1) could play a key role by degrading cytosolic DNA, which accumulates in tumors with genomic instability, thereby preventing activation of the cGAS‐STING pathway and the subsequent adaptive antitumor immune response. Consistently, they demonstrated that MSI‐H tumors exhibit elevated intrinsic TREX1 expression. Loss of TREX1 in those tumors results in tumor‐intrinsic type I interferon (IFN‐I) activation, increased infiltration of CD8^+^ T and natural killer (NK) cells in a dendritic cell (DC)‐dependent manner, and consequent tumor regression (Fig. 1). Conversely, IFN‐I treatment induces TREX1 expression, forming a negative feedback loop that dampens cGAS‐STING pathway activation. This tumor‐intrinsic mechanism ultimately contributes to immune evasion in MSI‐H tumors. Notably, this regulation can be reversed by ruxolitinib, which downregulates TREX1 through JAK/STAT pathway inhibition. They also demonstrated that the infiltrating CD8^+^ T cells displayed activation markers such as CD69, CD44, and CD28, together with reduced expression of exhaustion markers PD‐1 and T‐cell immunoglobulin and mucin‐domain containing‐3. Moreover, these tumor‐infiltrating T cells exhibited increased levels of granzyme B, IFN‐γ, TNF‐α, and IL‐2–STAT5 signaling, consistent with a robust cytotoxic and activated phenotype.

**Fig. 1:**
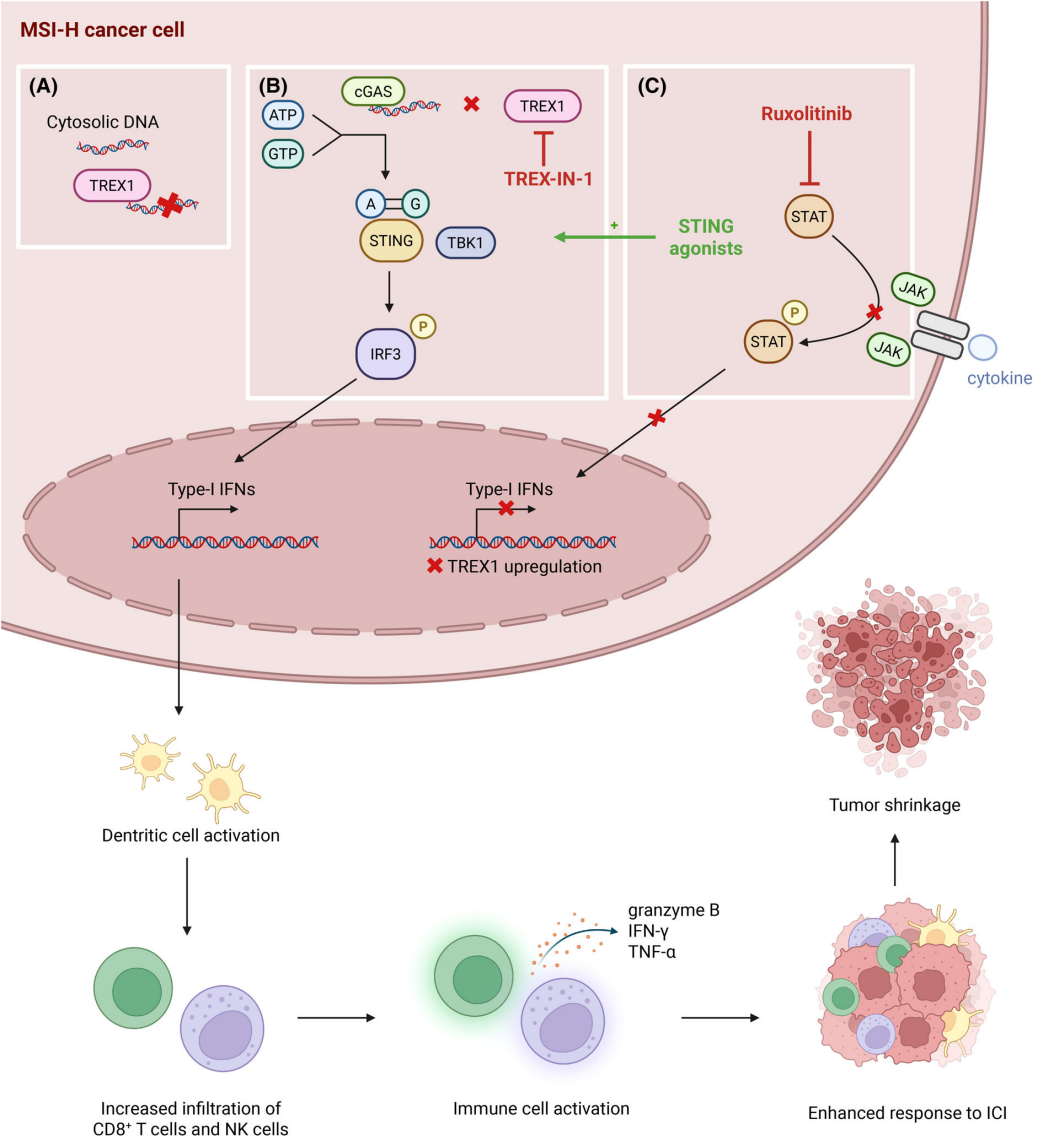
The cGAS–STING pathway in MSI‐H tumors: regulation by TREX1 and emerging therapeutic strategies to boost immunotherapy efficacy. (A). Cytosolic double‐stranded DNA can accumulate in MSI‐H tumors and activate the cGAS–STING pathway, leading to an antitumor immune response. TREX1 degrades cytosolic DNA and suppresses this activation. (B). TREX1 inhibitor (TREX‐IN‐1) prevents DNA degradation and promotes an increase of active CD8+ T cells and NK cells infiltration in a DC‐dependent manner, which in turn results in an enhanced response to immunotherapy. (C) Ruxolitinib (JAK1/2 inhibitor) preventing TREX1 upregulation and STING agonists are also being tested to enhance this pathway and improve responses to immune checkpoint inhibitors. ATP, Adenosine triphosphate; cGAS, cyclic GMP‐AMP synthase; GTP, Guanosine triphosphate; IFN, Interferon; IRF3, Interferon Regulatory Factor 3; JAK, Janus kinases; MSI‐H, high microsatellite instability; STAT, Signal Transducers and Activators of Transcription; STING, Stimulator of Interferon Genes; TBK1, TANK‐binding kinase 1; TNF, Tumor necrosis factors. Figure created in BioRender. https://BioRender.com/nr3b1vh

The results of this study suggest new therapeutic avenues for the treatment of MSI‐H tumors. TREX1 inhibition could be combined with existing immunotherapies to enhance patient responses and improve survival. The authors demonstrated that TREX1‐IN‐1 (specific inhibitor) suppresses tumor growth in MLH1‐deficient tumors by stimulating CD8^+^ T cell–mediated antitumor immunity. This study adds to a growing body of work exploring TREX1 as a potential modulator of immunotherapy and chemotherapy efficacy by enhancing T‐cell infiltration in cold tumors [9, 10, 11].

Although promising, TREX1‐IN‐1 has not yet been validated clinically. Ruxolitinib, a clinically approved JAK1/2 inhibitor for the treatment of myeloproliferative syndromes, to inhibit IFN‐I signaling and thereby prevent TREX1 upregulation in an MLH1‐dependent manner, could be used. The combination of nivolumab and ruxolitinib in 19 patients with Hodgkin lymphoma showed promising results with a best overall response rate of 53%, a lower infiltration in myeloid suppressive cells inside the tumor and an increase of activated T‐cell infiltration [12]. However, its benefit in solid tumors remains to be established. Xu *et al*. explored the possibility of modulating TREX1 expression through JAK/STAT pathway inhibition (e.g., with ruxolitinib) but they emphasized that IFN signaling remains essential for immune‐mediated tumor rejection in MSI‐H/dMMR tumors. Thus, while JAK inhibitors may have complex immunomodulatory effects in certain contexts, their use in MSI‐H tumors warrants cautious evaluation. Other strategies targeting different components of the cGAS–STING pathway, such as the development of STING agonists or modulators of downstream signaling molecules, are also being investigated in preclinical and clinical settings, further supporting the therapeutic potential of this pathway in cancer immunotherapy (Fig. 1). Yet, clinical responses have so far been modest, highlighting the need for improved delivery systems or combination strategies to fully exploit the therapeutic potential of this pathway [13, 14].

This study sheds light on the mechanisms underlying immune escape in MSI‐H tumors, highlighting the pivotal role of TREX1 in regulating the cGAS–STING pathway. These findings open new therapeutic perspectives aimed at modulating immune responses in MSI‐H cancers refractory to current immunotherapy approaches.

Nonetheless, MSI‐H tumors account for a minority of cancer cases, with most patients presenting MSS tumors. These tumors are characterized by a low TMB but display chromosomal instability, which has also been associated with the accumulation of genomic DNA in the cytosol. Therefore, collective efforts should be focused on elucidating the role of TREX1 and of the cGAS–STING pathway in this tumor subtype, which is crucial to extend the potential therapeutic benefit of immune checkpoint inhibitors to the larger group of patients who remain resistant to current immunotherapy strategies [15].

## Conflict of interest

The authors declare no conflict of interest.

## Author contributions

EB, JD, and MVdE have made a substantial, direct, and intellectual contribution to the work and approved it for publication.
